# Assessing of programmed cell death gene signature for predicting ovarian cancer prognosis and treatment response

**DOI:** 10.3389/fendo.2023.1182776

**Published:** 2023-06-05

**Authors:** Xin Lian, Bing Liu, Caixia Wang, Shuang Wang, Yuan Zhuang, Xiao Li

**Affiliations:** ^1^ Department of Otolaryngology Head and Neck Surgery, Shengjing Hospital of China Medical University, Shenyang, China; ^2^ Department Obstetrics and Gynecology, Shengjing Hospital of China Medical University, Shenyang, China

**Keywords:** programmed cell death, ovarian cancer, prognosis, chemotherapeutic drug selection, immunotherapy

## Abstract

**Background:**

Programmed cell death (PCD) is an overwhelming factor affecting tumor cell metastasis, but the mechanism of PCD in ovarian cancer (OV) is still uncertain.

**Methods:**

To define the molecular subtypes of OV, we performed unsupervised clustering based on the expression level of prognosis related PCD genes in the Cancer Genome Atlas (TCGA)-OV. COX and least absolute shrinkage and selection operator (LASSO) COX analysis were used to identify the OV prognostic related PCD genes, and the genes identified according to the minimum Akaike information criterion (AIC) were the OV prognostic characteristic genes. According to the regression coefficient in the multivariate COX analysis and gene expression data, the Risk Score of OV prognosis was constructed. Kaplan-Meier analysis was conducted to assess the prognostic status of OV patients, and receiver operating characteristic (ROC) curves were conducted to assess the clinical value of Risk Score. Moreover, RNA-Seq date of OV patient derived from Gene Expression Omnibus (GEO, GSE32062) and the International Cancer Genome Consortium (ICGC) database (ICGC-AU), verifying the robustness of the Risk Score *via* Kaplan-Meier and ROC analysis.Pathway features were performed by gene set enrichment analysis and single sample gene set enrichment analysis. Finally, Risk Score in terms of chemotherapy drug sensitivity and immunotherapy suitability was also evaluated in different groups.

**Results:**

9-gene composition Risk Score system was finally determined by COX and LASSO COX analysis. Patients in the low Risk Score group possessed improved prognostic status, immune activity. PI3K pathway activity was increased in the high Risk Score group. In the chemotherapy drug sensitivity analysis, we found that the high Risk Score group might be more suitable for treatment with PI3K inhibitors Taselisib and Pictilisib. In addition, we found that patients in the low-risk group responded better to immunotherapy.

**Conclusion:**

Risk Score of 9-gene composition of PCD signature possesses promising clinical potential in OV prognosis, immunotherapy, immune microenvironment activity, and chemotherapeutic drug selection, and our study provides the basis for an in-depth investigation of the PCD mechanism in OV.

## Introduction

Ovarian cancer (OV) in females is challenging to diagnose in a timely manner due to the challenges of clinical sampling and the lack of early typical symptoms (1), as the ovaries are deep in the pelvis and small in size. In consequence, OV remains one of the deadliest causes of cancer death in females worldwide, with more than 300,000 cases of OV and 190,000 deaths (2) currently. Frustratingly, approximately 70% of OV patients treated with conventional surgery and platinum-based chemotherapy recur or develop chemoresistance, with 5-year survival rate of less than 40% (3). Considering the extremely poor prognostic status of OV, effective prognostic signatures and the development of innovative therapeutic targets are urgent to improve OV survival rates.

Programmed cell death (PCD) is a genetically dominated mode of cellular normal death (4). Currently, 12 PCD mechanisms were investigated and confirmed, which include, apoptosis, necroptosis, ferroptosis, pyroptosis, netotic cell death, entotic cell death, lysosome-dependent cell death, parthanatos, autophagy-dependent cell death, oxeiptosis, and alkaliptosis and cuproptosis, cuproptosis was the recently revealed PCD pathway (5, 6). Studies revealed confirmed that PCD was the essential factor for tumor cells capable of developing as well as metastasizing; in normal environment, cells are unable to replicate and metastasize permanently, and when PCD is inhibited, the normal way of cell death is dysregulated, which in turn develops into cancer cells; therefore, overcoming the PCD mechanism is the fundamental cause of tumor cell formation (7). According to the relevance of PCD for cancer development, several theories of relevant targeted therapeutic approaches were proposed. For example, BCL-2 inhibitors modulate the apoptotic process and were approved by the FDA as new therapeutic options for lymphoma (8). The GSDME mechanism-based approach to cell scorching was novel immunotherapeutic therapy (9). The transformation between pyroptosis and apoptosis was also current topical direction in the attempt (10). A comprehensive summary of the current PCD mechanism in OV remains incomplete, and the detailed function of PCD in OV is thinly investigated.

In this study, we successfully constructed a prognostic Risk assessment algorithm (Risk Score) for OV patients. According to our results, Risk Score was also significantly correlated with the immune microenvironment status and chemotherapy/immunotherapy sensitivity of OV patients, which could guide the personalized treatment of cancer patients.

## Materials and methods

### Data acquisition

Somatic mutation data, clinical data, and RNA-Seq data of OV samples were obtained from The Cancer Genome Atlas (TCGA, https://portal.gdc.cancer.gov/) database (TCGA-OV, FPKM). The Gene Expression Omnibus (GEO, https://www.ncbi.nlm.nih.gov/gds) database was searched for “Ovarian cancer”, and RNA-Seq data and clinical data for GSE32062 were obtained. The ICGC-AU dataset was also extracted from The International Cancer Genome Consortium (ICGC, https://dcc.icgc.org/). In this study, TCGA-OV was considered as the training set, and GSE32062 and ICGC-AU were considered as the validation set. 1078 PCD-correlated genes were acquired from a previous research (11).

### Data pre-processing

RNA-Seq data were processed through the sangerbox website (http://sangerbox.com/home.html) (12) Samples with incomplete clinical information with survival times<30 days were eliminated, and normal samples were removed and only tumor samples were retained. The number of OV samples with complete clinical data in TCGA-OV, GSE32062 and ICGC-AU were 363, 260, and 93. Samples with missing somatic mutation data were removed and 428 samples in TCGA-OV with complete somatic mutation data were retained during the analysis of somatic data.

### Identification of molecular subtypes of PCD

Univariate COX analysis was conducted to determine prognosis-related PCD-related genes. Consistent cluster analysis was then conducted on samples in TCGA-OV based on the expression matrix of the above genes to obtain PCD molecular subtypes (13) following the method of Wilkerson et al. According to the “pam” algorithm and “pearson” as the metric distance, we performed 500 bootstraps, and each bootstraps procedure included 80% of the training set patients. The number of clusters was set from 2 to 10, and the optimal number of clusters was determined based on the consistency matrix and the consistency cumulative distribution function.

### Differential expression analysis and functional enrichment analysis

To address the molecular subtypes of PCD, differential expression analysis of subtypes was conducted through the limma package (14) to access the differentially expressed genes (DEGs) in subtypes. To explore the biological functions as well as pathways involved in DEGs, Gene Ontology (GO) and Kyoto Encyclopedia of Genes and Genomes (KEGG) enrichment analysis (FDR<0.05) was conducted by clusterProfiler package (14).

### Construction of risk score

Univariate COX analysis was performed for all DEGs in the subtype and genes influencing OV prognosis were identified (p<0.05). Then LASSO COX analysis was further performed by glmnet package (15) and low similarity and more significantly genes were retained. Finally, genes that markedly affected OV prognosis were obtained by multivariate COX analysis. Based on the corresponding regression coefficients and expression data of the signature genes, we constructed the Risk Score and calculated it by the following equation.


$$RiskScore=\sum coefi_{i}*expression\;i$$

In the formula, coef and expression indicated the regression coefficient and expression data of genes.

### Assessment and validation of risk score

The surv_cutpoint() function in the survminer package was conducted to group samples in TCGA-OV. According to the Risk Score, the surv_cutpoint() function evaluated the likelihood of all potential groups. When the outcome variable was “time to event”, the threshold at this time was considered to be the best grouping threshold (16). According to the optimal grouping threshold determined by survminer, in the TCGA-OV cohort, the Risk Score>grouping threshold was defined as the high Risk Score group, and the Risk Score<grouping threshold was defined as the low Risk Score group. Kaplan-Meier model was constructed to assess median survival time and overall survival time in both groups. ROC curves were plotted and Risk Score prediction power was determined based on 1-, 3-, and 5-year AUCs. Validation was performed in GSE32062 and ICGC-AU.

### Functional analysis

For biological pathways with discrepancies in molecular subtypes and Risk Score groups. HALLMARK gene sets were accessed from The Molecular Signatures Database (MSigDB, https://www.gsea-msigdb.org/gsea/msigdb/) database database. Gene set enrichment analysis (GSEA) was conducted for groups (17). Single sample Gene Set Enrichment Analysis (ssGSEA) was implemented through the GSVA package (18) and was designed to identify biological pathways activated/repressed in groups. Cancer-related pathway activity was determined by PROGENy algorithm (19).

### Evaluation of tumor microenvironment

For infiltrating immune cell abundance, stromalscore, and tumor purity in the tumor microenvironment (TME), the ESTIMATE algorithm (20) was performed to assess TME activity in OV patients, and the CIBERSORT algorithm (21) was performed to assess the infiltrating abundance of 22 immune cell types in OV patients. The 7 steps of cancer immune cycle (step 1: releasing cancer cell antigens, step 2: cancer antigen presentation, step 3: priming and activation, step 4. trafficking immune cells to tumors, step 5: infiltration of immune cells into tumors, step 6: recognition of cancer cells by T cells, step 7: killing cancer cells) of the characteristic gene sets, and the cancer immune cycle activity in the groups was assessed by the ssGSEA method.

### Response to immunotherapy

Immune checkpoint genes were obtained from Hu et al. A total of 79 genes (22) were examined for the expressions of these immune checkpoint genes in OV samples. The Myeloid-derived suppressor cells (MDSC), Dysfunction Score, TIDE Score, Tumor-Associated Macrophages M2 (TAM.M2) Score, Exclusion Score, Cancer-associated fibroblast (CAF) Score of OV samples were obtained from the Tumor Immune Dysfunction and Exclusion (TIDE, http://tide.dfci.harvard.edu/) database to determine immunotherapy response.

### Sensitivity to chemotherapy

OV cell expression data of 70 chemotherapy drug treatments in OV patients were obtained from the Genomics of Drug Sensitivity in Cancer (GDSC, https://www.cancerrxgene.org/) database, and the oncoPredict package (23) was conducted to calculate the half-maximal inhibitory concentration (IC50) data of drugs to compare drug IC50 differences in Risk Score groups, and the Spearman correlation among Risk Score, prognostic signature genes and drug IC50 was compared to explore the most adaptable chemotherapy for patients.

### Statistical analysis

The R packages employed in this study were obtained from the Bioconductor R project (24) and data analysis was performed by R software (version: 4.1.1). sangerbox contributed to data pre-processing. p<0.05 was considered statistically significant for all statistical tests.

## Results

### Variation landscape of programmed cell death-related genes in OV patients

**Figure 1:**
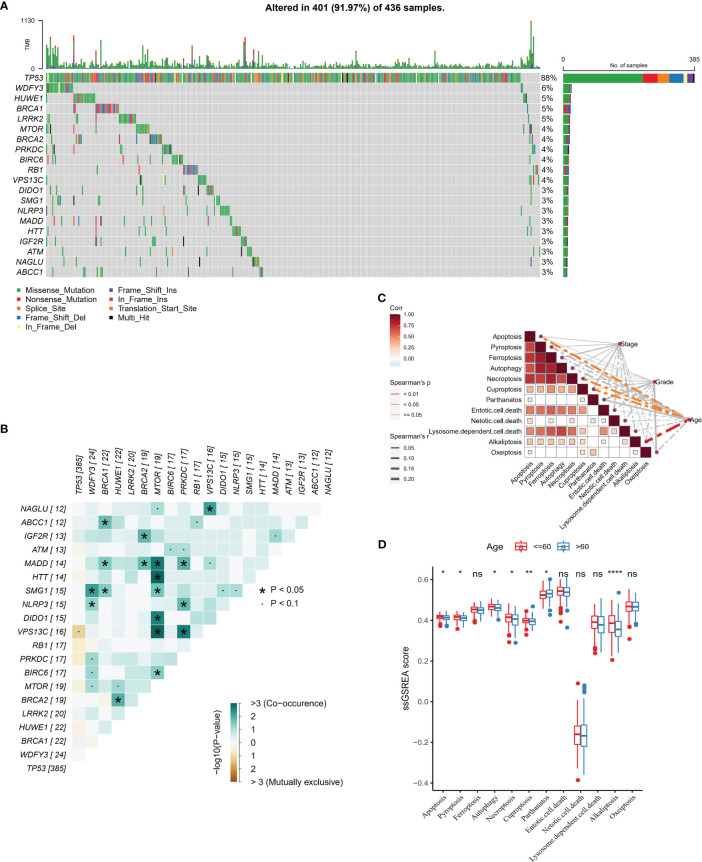
PCD-related gene mutation landscape **(A)** Top 20 gene mutation landscape waterfall map **(B)** Mutual exclusivity and co-occurrence phenomenon correlation heat map among Top 20 genes **(C)** PCD signature ssGSEA score is correlated with Stage, Grade, and Age Sex heat map **(D)** PCD signature ssGSEA score in Age group. * for p < 0.05, ** for p < 0.001,*** for p < 0.0001, **** for p < 0.00001, and ns for no significance, p > 0.05.

In this study, the number of samples with mutation data in TCGA-OV was 428 cases. First, we analyzed the molecular landscape of mutations in programmed cell death-related genes in the TCGA-OV samples. We observed that 401 (98.2%) OV samples possessed top 20 mutations, among which the top 3 mutated genes were TP53 (88%), WDFY3 (6%), and HUWE1 (5%) (**Figure 1A**). We also found that co-occurrence was occurred more frequently between top 20 genes, and there were remarkable co-occurrence between MTOR with MADD, HTT, SMG1, DIDO1, VPS13C, and BIRC6 (**Figure 1B**). Furthermore, by ssGSEA analysis, we evaluated 12 PCD signature ssGSEA scores in those samples and analyzed the spearman correlation among them and Stage, Grade, and Age. The results showed that apoptosis, necroptosis, cuproptosis, and alkaliptosis showed negative significant correlations (spearman’s p< 0.01) with Age (**Figure 1C**). Further, we found higher Apoptosis, Pyroptosis, Autophagy, Necroptosis, Cuproptosis, Alkaliptosis signature ssGSEA scores in the lower age group (age<= 60) (**Figure 1D**).

### Molecular subtypes of programmed cell death-related genes

**Figure 2:**
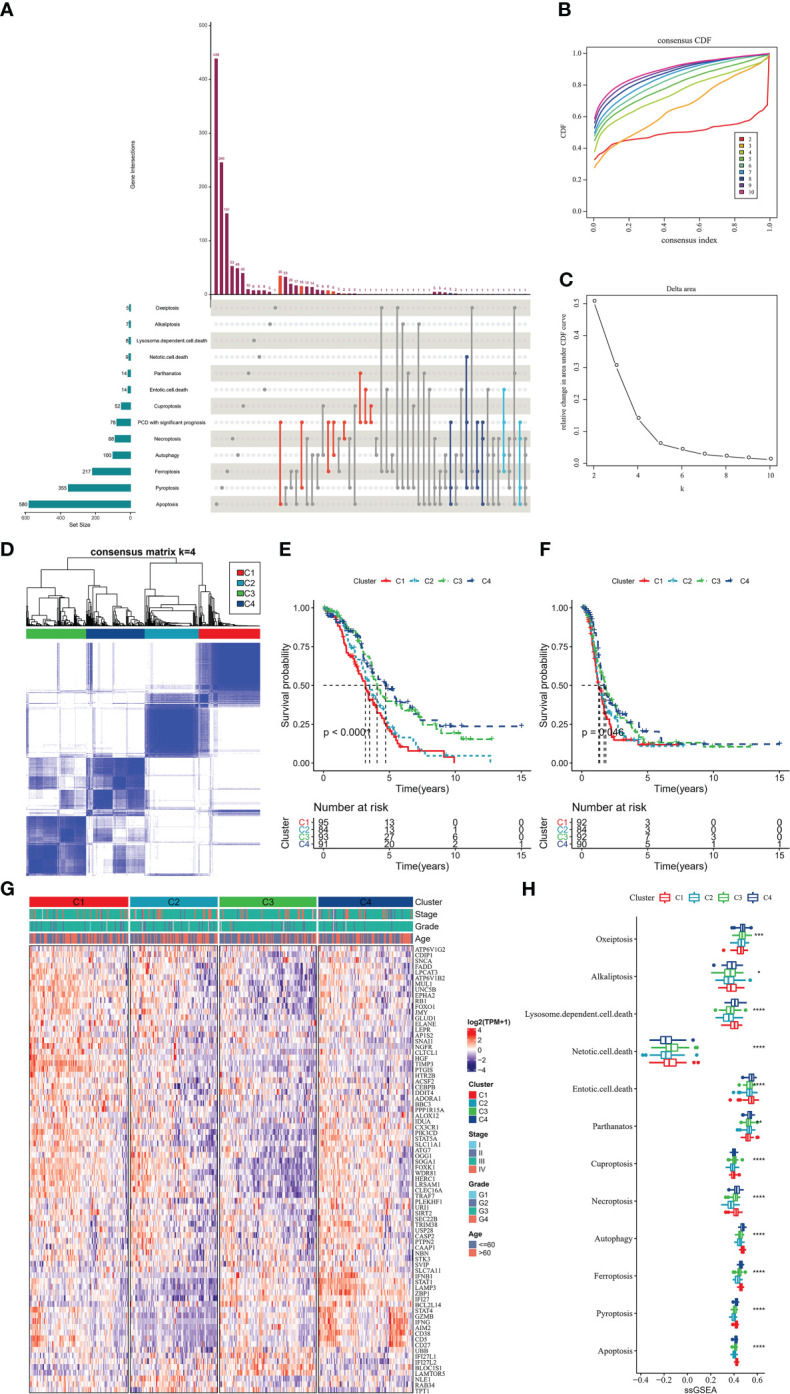
PCD molecular subtype **(A)** upset plot showing the distribution of prognosis-related genes in 12-PCD **(B)** cumulative distribution function **(C)** CDF Delta area curve **(D)** sample clustering heat map when k=4 **(E)** OS curves of 4 molecular subtypes **(F)** PFS curves of 4 molecular subtypes **(G)** prognostic-related PCD-related gene expression heat map **(H)** 12 PCD signature ssGSEA score in 4 molecular subtypes. * for p < 0.05, ** for p < 0.001,*** for p < 0.0001, **** for p < 0.00001, and ns for no significance, p > 0.05.

In this study, we obtained 363 samples with corresponding clinical information and expression data from the TCGA-OV cohort. Univariate COX analysis confirmed that 78 of 1078 PCD-related genes were markedly correlated with OV patients’ prognosis (**Supplementary Figure S1**), and the prognostic genes contained 40 apoptosis genes, 22 pyroptosis genes, 10 ferroptosis genes, 10 autophagy genes, and 10 necroptosis genes (**Figure 2A**). Then, according to the 78-gene expression matrix, the results of the consistency clustering analysis of 363 OV samples showed that these patients could be classified into four subtypes (C1, C2, C3, C4) (**Figures 2B–D**). Compared with C1, C2, and C3 patients, OV patients in C4 experienced better OS and PFS (**Figures 2E, F**). 78-gene expression levels and the distribution of Stage, Grade, and Age information in the four subtypes were demonstrated in **Figure 2G**, we found that prognostic related PCD genes showed more active expression levels in the C1 subtype. The 12 PCD signature ssGSEA scores were remarkably distinct in all four subtypes, with the majority scoring higher in C1 and C4 (**Figure 2H**).

### Investigation of the immune microenvironment of molecular subtypes

**Figure 3:**
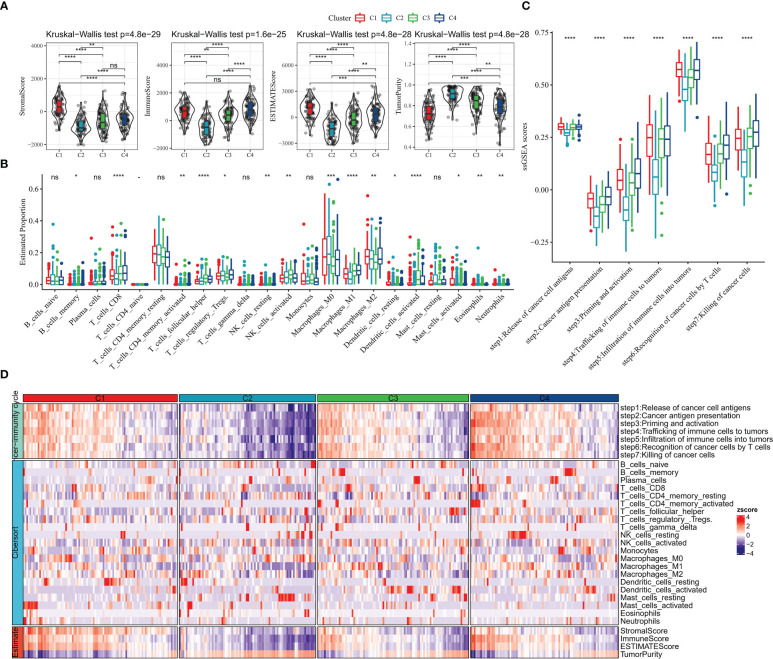
TME activity in molecular subtypes **(A)** ESTIMATE result **(B)** CIBERSORT result **(C)** 7-cancer immune cycle signature ssGSEA score **(D)** heat map showing ESTIMATE, CIBERSORT, 7-cancer immune cycle difference. * for p < 0.05, ** for p < 0.001,*** for p < 0.0001, **** for p < 0.00001, and ns for no significance, p > 0.05.

We further explored the TME status of the 4 subtypes of patients.ESTIMATE results showed that C1 patients had the highest StromalScore, ESTIMATEScore, and the lowest TumorPurity (**Figure 3A**). Further, the relative infiltration abundance of 22 immune cells in each group was analyzed, and C4 patients had higher abundance of Macrophages_M1, T_cells_CD8, T_cells_regulatory_Tregs, T_cells_follicular_helper (**Figure 3B**). The ssGSEA enrichment analysis was performed with genes characteristic of the 7 stages of the cancer immune cycle to explore the differences between the tumor immune cycle activities of patients with the 4 subtypes. ssGSEA scores for all 7 stages of the C2 subtype were remarkably lower than those of the other subtypes, whereas compared with those of the C2 and C3 subtypes, ssGSEA scores for the 7 stages of the C1 and C4 subtypes were significantly higher (**Figure 3C**). Finally, we drew a heat map to show the profile of tumor immune cycle score, immune cell score and ESTIMATE score in all samples in C1, C2, C3 and C4. Overall, patients with C1 exhibited higher StromalScore and ESTIMATEScore, low levels of TumorPurity, and active tumor immune cycle activity (**Figure 3D**).

### TIDE and TMB analysis suggests immunotherapy potential benefits for molecular subtypes

**Figure 4:**
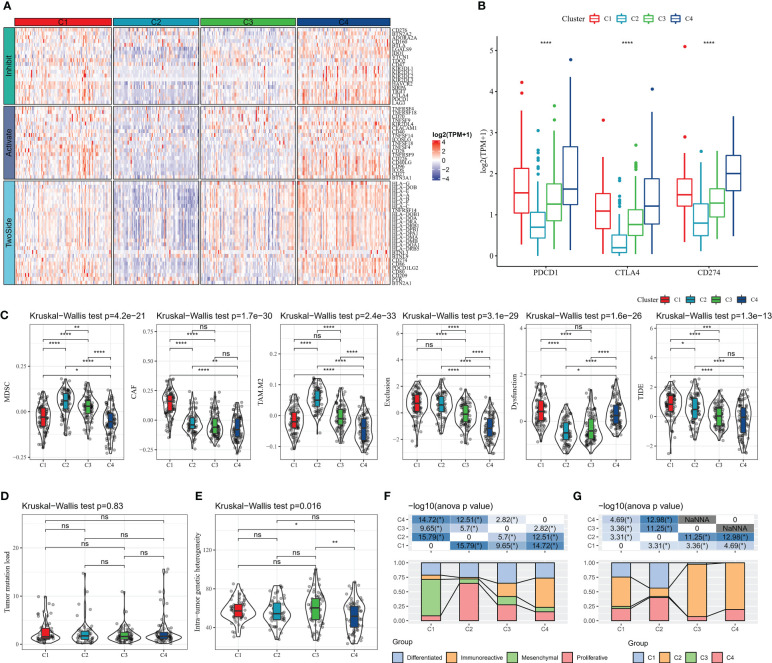
Relationship between molecular subtypes and immunotherapy **(A)** Immune checkpoint gene expression heat map **(B)** PDCD1 (PD-1), CTLA4, CD274 (PD-L1) expression **(C)** TIDE results **(E)** Intra-tumor Genetic heterogeneity **(F)** molecular subtype comparison. * for p < 0.05, ** for p < 0.001,*** for p < 0.0001, **** for p < 0.00001, and ns for no significance, p > 0.05.

Immune checkpoint blockade (ICB) therapy that inhibits the expression of immune checkpoint is an emerging option in cancer treatment. We evaluated the expression levels of 79 immune checkpoints in patients with four subtypes, and it was clearly observed that the expression levels of immune checkpoint were lower in C2 and C3 subtypes, higher in C1 and C4 subtypes, and highest in C4 (**Figure 4A**). The current clinically approved ICB drugs are mainly PDCD1 (PD-1), CTLA4, CD274 (PD-L1), and the results showed that the highest expression levels of CTLA4, PDCD1 (PD-1), CD274 (PD-L1) were found in C4 patients (**Figure 4B**). We also observed that patients with C1 subtype had high levels of CAF score, Exclusion score, Dysfunction score, and TIDE score, and patients with C4 subtype had the lowest levels of MDSC score, CAF score, TAM.M2 score, Exclusion score, and Dysfunction score, and TIDE score, suggesting that C4 patients were more sensitive to ICB treatment and had a higher likelihood of benefit (**Figure 4C**). Additionally, the TMB of C4 subtype was found to be slightly higher than other subtypes, but there was no statistical difference (**Figure 4D**). In contrast, there was no remarkable difference in Intra-tumor genetic heterogeneity among the four subtypes (**Figure 4E**). According to the molecular subtype study of OV in the pan-cancer molecular landscape by Thorsson et al, the majority of patients in C1 defined in this study belonged to the Mesenchymal subtype and the majority of patients in C4 belonged to the Immunoreactive (**Figure 4F**). In contrast, according to Thorsson et al. molecular subtypes based on immune signatures, a higher proportion of patients with C2 immune subtypes in C3 and C4 (**Figure 4G**). Overall, the molecular subtypes of PCD we defined are complementary to the new molecular characterization typing of OV and enrich the existing clinical typing criteria.

### Pathway activity among PCD molecular subtypes

**Figure 5:**
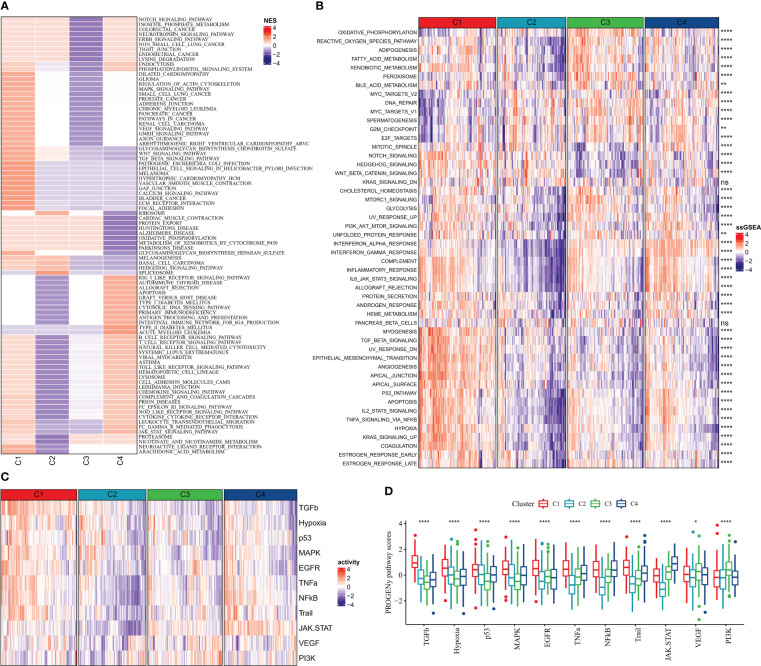
Pathway activity of molecular subtypes in China **(A)** GSEA **(B)** ssGSEA **(C)** Heat map showing cancer-related pathway activity **(D)** Cancer-related pathway activity boxplot. * for p < 0.05, ** for p < 0.001,*** for p < 0.0001, **** for p < 0.00001, and ns for no significance, p > 0.05.

According to GSEA enrichment analysis, PATHWAYS_IN_CANCER, WNT_SIGNALING_PATHWAY, TGF_BETA_SIGNALING_PATHWAY, MAPK_SIGNALING_PATHWAY, VEGF_SIGNALING_PATHWAY in C1 subtype which were EMT-related pathways were activated, and TYPE_I_DIABETES_MELLITUS, T_CELL_RECEPTOR_SIGNALING_PATHWAY, B_CELL_RECEPTOR_SIGNALING_PATHWAY, which were immune-related pathways were activated in C4 subtype (**Figure 5A**). According to ssGSEA functional analysis, 48 KEGG pathways were remarkably different among the four subtypes (kruskal.test, p<0.05), and C1 and C4 subtypes were mainly enriched in immune-related pathways, for example, INFLAMMATORY_RESPONSE, INTERFERON_ALPHA_RESPONSE, and IL6_JAK_STAT3_SIGNALING, INTERFERON_GAMMA_RESPONSE, it was notable that the scores of these pathways were higher in C4 subtypes (**Figure 5B**). Differences in the activities of oncogenic-related pathways were examined in the four molecular subtypes, TGFb, Hypoxia, p53, MAPK, EGFR, TNFa, NFkB, Trail, JAK.STAT, VEGF, and PI3K pathway activities were assessed by the PROGENy algorithm. We found that TGFb, Hypoxia, p53, MAPK, EGFR, TNFa, and Trail pathway activity scores were significantly higher in C1 than in C2, C3, and C4 subtypes (**Figures 5C, D**), indicating that patients with C1 subtype had high cancer cell activity and increased risk of metabolism and metastasis, which promoted cancer progression.

### Functional analysis of differentially expressed genes in molecular subtypes of PCD

**Figure 6:**
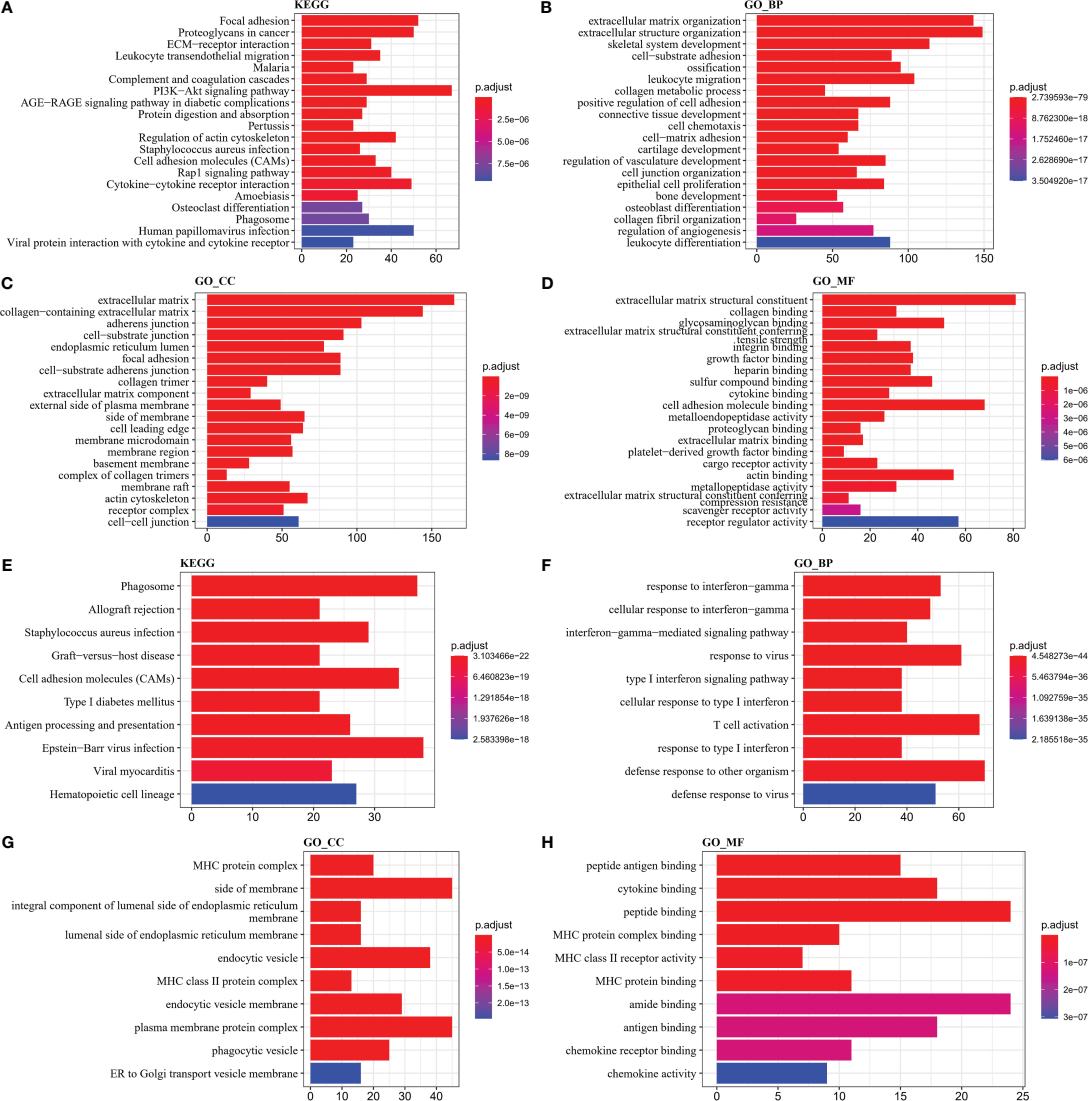
GO and KEGG enrichment analysis **(A–D)** KEGG, Biological Process, Cellular Component, Molecular Function results in C1 **(E–H)** KEGG, Biological Process, Cellular Component, Molecular Function results in C4.

To investigate the reasons for the differences in the activity of the four subtypes of pathways, we identified DEGs in the groups and performed functional analysis. First, the “C1_VS_C2&C3&C4” (C1), “C2_VS_C1&C3&C4” (C2), “C3_VS_C1&C2& C4” (C3), and “C4_VS_C1&C2&C3” (C4) were analyzed for differences. 354 DEGs (4 down-regulated and 350 up-regulated) were identified in the C1 group, 300 DEGs (277 down-regulated and 23 up-regulated) were identified in the C2 group, and 82 DEGs were identified in the C3 group identified 82 DEGs (11 up-regulated and 71 down-regulated), and 90 DEGs (79 up-regulated and 11 down-regulated) in the C4 group (FDR< 0.05, |log2FC| > log2(1.5)) (**Supplementary Figure S2**). Functional analysis was then performed for upregulated DEGs in C1 and C4 groups. By KEGG analysis, ECM-receptor interaction, PI3K-Akt signaling pathway, Cytokine-cytokine receptor interaction were the major upregulated biological pathways in the C1 group, and Phagosome, Cell adhesion molecules (CAMs), and Epstein-Barr virus infection were the major upregulated biological pathways in the C4 group (**Figures 6A**, **E**). According to GO analysis, an increase in extracellular matrix, collagen containing extracellular matrix, and an improvement in extracellular matrix structural constituent and extracellular matrix structural constituent conferring increased extracellular matrix organization and extracellular structure organization in C1 group (**Figures 6B–D**). The increase of MHC class II protein complex, MHC protein complex, and the enhancement of cytokine binding, MHC protein complex binding, and MHC protein binding, MHC class II receptor activity led to type I interferon signaling pathway, cellular response to type I interferon, T cell activation, response to type I interferon upregulation of these functions (**Figures 6F–H**). These findings also further validated that the TME activity in the C4 subtype was higher than that in other subtypes.

### Establishment and evaluation of risk score

**Figure 7:**
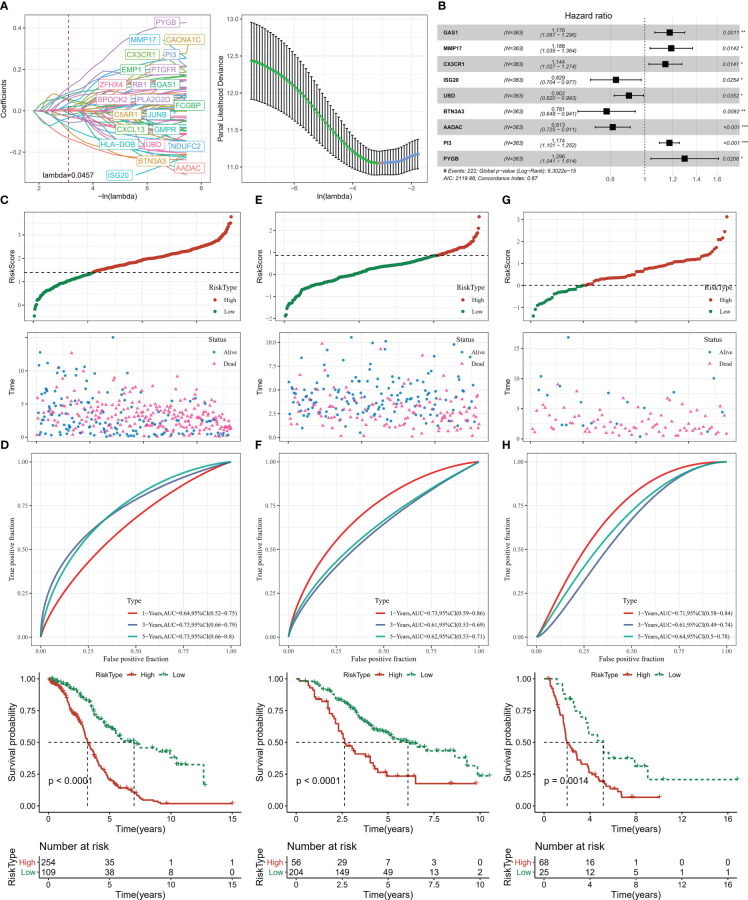
Risk Score construction and verification **(A)** LASSO COX analysis **(B)** 9-gene forest plot **(C, D)** Risk Score distribution, ROC curve, Kaplan-Meier curve of patients in TCGA-OV **(E, F)** Risk Score distribution of patients in GSE32062, ROC curve, Kaplan-Meier curve **(G, H)** Risk Score distribution of patients in ICGC-AU, ROC curve, Kaplan-Meier curve. * for p < 0.05, ** for p < 0.001,*** for p < 0.0001, **** for p < 0.00001.

Univariate COX analysis was performed on DEGs in groups C1, C2, C3 and C4 and 67 genes affecting OV prognosis were identified, of which 48 genes with HR > 1 were considered risk factors and 19 genes with HR< 1 were considered protective factors. The high number of genes in the model will increase the operational complexity as well as the accuracy of the clinical test. Further LASSO COX analysis of the 67 genes affected OV prognosis was performed to reduce the number of genes with high similarity but low weight in the model, and the results showed that 23 significant low similarity genes were identified (**Figure 7A**). Finally, 9 genes were identified as PCD-related genes affecting OV prognosis, with HR>1 for GAS1, MMP17, CX3CR1, PI3, and PYGB as risk factors and HR<1 for ISG20, UBD, BTN3A3, and AADAC as protective factors (**Figure 7B**). Based on the COX regression coefficients of the 9-gene and the expression data, we constructed the Risk Score, a prognostic assessment system for OV based on PCD signature, with the specific formula Risk Score=+0.162*GAS1 + 0.172*MMP17 + 0.134*CX3CR1-0.187*ISG20-0.103*UBD-0.247*BTN3A3- 0.208*AADAC+0.16*PI3 + 0.259*PYGB.

Next, in order to evaluate the Risk Score as a prognostic assessment parameter, patients’ survival status, ROC curves, and Kaplan-Meier survival curves were assessed in the training set (TCGA-OV) and validation set (GSE32062, ICGC-AU). Based on the optimal grouped Risk Score thresholds in the survminer package, OV patients in the three datasets were established as high Risk Score and low Risk Score groups, and it was observed that there was gradual increase in deaths over time and with higher Risk Scores (**Figures 7C, E, G**). Kaplan-Meier analysis showed that median and overall survival was better in the low Risk Score group. The ROC curves for all three data sets also showed an AUC value for Risk Score predicting survival at 1, 3 and 5 years greater than 0.6 (**Figures 7D, F, H**). Risk Score could serve as a tool for OV prediction.

### Surveys of clinical information in risk score groups

**Figure 8:**
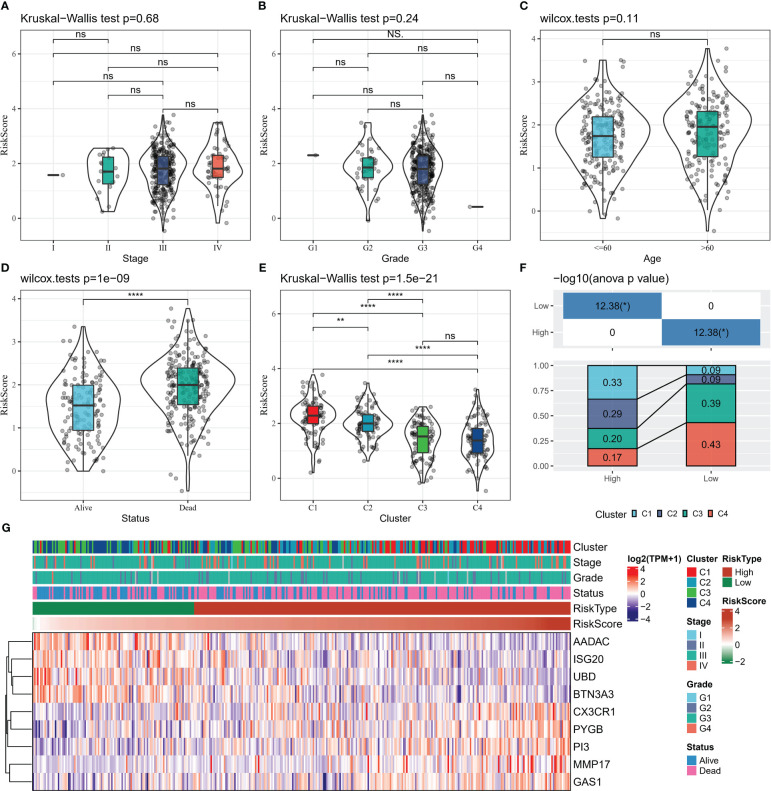
Relationship between clinical data and Risk Score **(A–D)** Risk Score distribution in Stage, Grade, Age, Status grouping **(E)** Risk Score distribution in four subtypes **(F)** Proportion of patients in four subtypes in Risk Score grouping Statistical analysis **(G)** heat map shows 9-gene expression heat map in Stage, Grade, Age, Status, Risk Score groups. * for p < 0.05, ** for p < 0.001,*** for p < 0.0001, **** for p < 0.00001, and ns for no significance, p > 0.05.

The distribution of Risk Scores of patients in different clinicopathological subgroups was analyzed. We found no remarkable differences in Risk Score between the Stage, Grade and age groups (**Figures 8A–C**). Compared to surviving patients, death patients had considerably higher Risk Scores (p<0.0001) (**Figure 8D**). In contrast, the highest Risk Score was found in C1 and the lowest in C4 among the 4 subtypes of patients (**Figure 8E**). The proportion of patients with the four subtypes in the Risk Score subgroup was calculated, with a higher proportion of patients with good prognosis in the C3 and C4 subtypes in the low Risk Score group and a higher proportion of patients with poor prognosis in the C1 and C2 subtypes in the high Risk Score group (**Figure 8F**). From the heat map of 9-gene combined with clinical information in the subgroup of patients, the expression levels of risk factors GAS1, MMP17, CX3CR1, PI3, and PYGB were found to be upregulated with increasing Risk Score, while the expression levels of protective factors ISG20, UBD, BTN3A3, and AADAC decreased with increasing Risk Score (**Figure 8G**).

### Survey of TME activity in risk score groups

**Figure 9:**
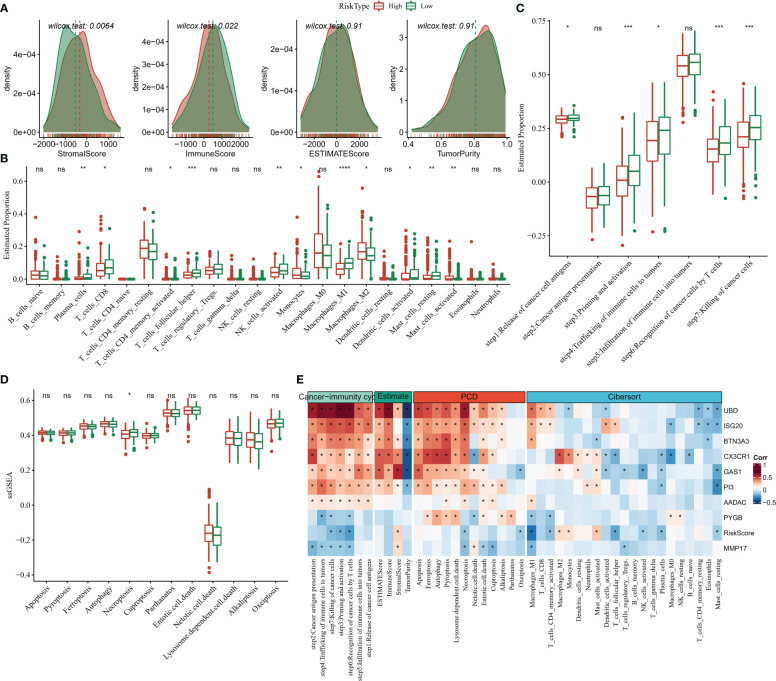
TME activity in Risk Score grouping **(A)** ESTIMATE **(B)** CIBERSORT **(C)** 7-cancer immune cycle signature ssGSEA score **(D)** 12 PCD signature ssGSEA score **(E)** heat map showing Risk Score and ESTIMATE, CIBERSORT, 7- Correlation between cancer immune cycle signature ssGSEA score and 12 PCD signature ssGSEA score. * for p < 0.05, ** for p < 0.001,*** for p < 0.0001, **** for p < 0.00001, and ns for no significance, p > 0.05.

Compared to the high Risk Score group, we found that patients in the low Risk Score group had higher ImmuneScore and lower StromalScore (**Figure 9A**), suggesting that the immune system was more active in the low Risk Score group and that there was less activity of pro-cancer related molecules in the stromal cells, which in turn increased their survival. We further observed higher abundance of cancer-suppressing T_cells_CD8, immune cells, Macrophages_M1, NK_cells_activated in the low Risk Score group and higher pro-cancer immune cell fraction Macrophages_M2 in the high Risk Score group (**Figure 9B**). The low Risk Score group also showed stronger cancer immune cycle activity (**Figure 9C**). In addition, we also compared the 12 PCD signature ssGSEA score differences in the Risk Score subgroups and showed that the low Risk Score group was significantly enriched in Necroptosis (**Figure 9D**). Finally, spearman correlations between 9-gene expression levels and TME activity, immune cell infiltration abundance, and cancer immune cycle activity were also assessed, with heat map showing significant correlations between UBD, ISG20, CX3CR1 and the cancer immune cycle, multiple programmed death modalities associated, and multiple immune cells (**Figure 9E**). These results demonstrated that the low Risk Score group had better immune activity and tumour-associated immune cell fractions, suggesting a more active immune system and better prognosis for the low Risk Score group.

### Immunotherapy sensitivity

**Figure 10:**
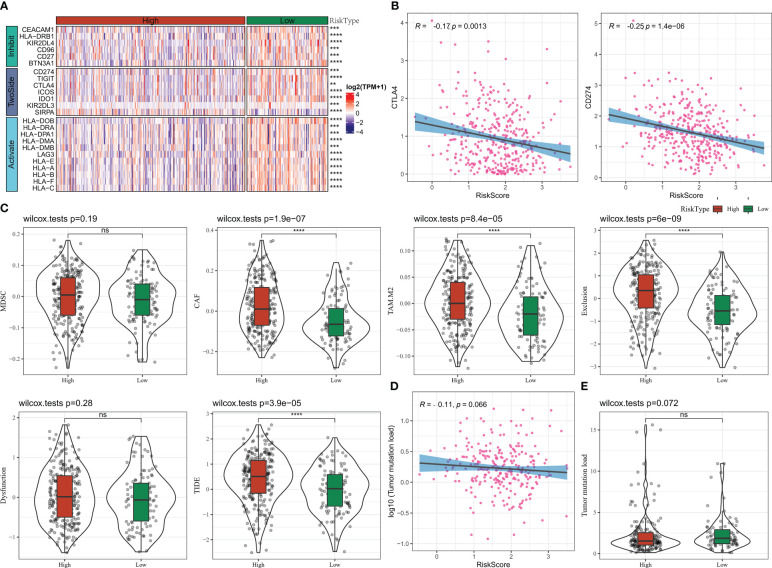
Risk Score group immunotherapy analysis **(A)** Immune checkpoint gene expression heat map **(B)** RiskScore and CTLA4, CD274 correlation **(C)** TIDE results **(D)** TMB and Risk Score correlation **(E)** TMB statistics. * for p < 0.05, ** for p < 0.001,*** for p < 0.0001, **** for p < 0.00001, and ns for no significance, p > 0.05.

In this study, we compared the differences in immune checkpoint gene expression among the Risk Score subgroups. 24 differentially expressed immune checkpoint genes were identified, with most of them being significantly more highly expressed in the low Risk Score group (**Figure 10A**). We further found a significant negative correlation between Risk Score and CTLA4 and CD274 (PD-L1) expression (**Figure 10B**). We also analysed the difference in response to immunotherapy benefit in the Risk Score grouping. The CAF score, TAM.M2 score, Exclusion score and TIDE score were lower in the low Risk Score compared to the high Risk Score group, suggesting that the lower the Risk Score, the lower the occurrence of immune escape (**Figure 10C**). The lower the Risk Score, the lower the immune escape and the higher the ICB treatment sensitivity (**Figure 10C**). Furthermore, Risk Score was negatively correlated with TMB, with TMB being higher in the low Risk Score group than in the high Risk Score group, but the difference was not statistically significant (**Figures 10D, E**). These findings suggested that the Risk Score could be used as potential biomarkers for the population for which ICB therapy was prescribed.

### Chemotherapy drug resistance susceptibility

**Figure 11:**
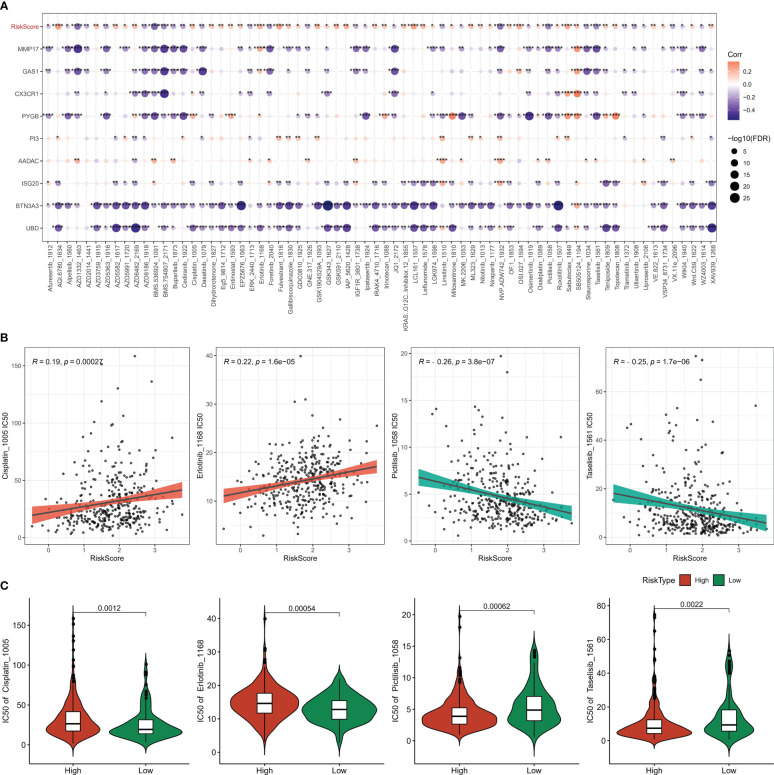
Chemotherapy sensitivity analysis **(A)** heat map of the association between Riskscore, genes, and sensitivity to 70 drugs **(B)** correlation between Risk Score and Pictilisib, Taselisib, Cisplatin, Erlotinib sensitivity **(C)** IC50 statistics of Pictilisib, Taselisib, Cisplatin, Erlotinib in the Risk Score groups. * for p < 0.05, ** for p < 0.001,*** for p < 0.0001, **** for p < 0.00001, and ns for no significance, p > 0.05.

To explore the relationship between the model and drug susceptibility, the Risk Score was assessed in relation to the IC50 of chemotherapeutic drugs. The results showed that the IC50 of 70 drugs was remarkably correlated with the Risk Score (**Figure 11A**). In addition, we found higher IC50s for cisplatin and erlotinib and lower IC50s for pictetinib and taselisib in the high-risk score group, suggesting that OV patients in the high-risk score group are more likely to develop resistance to standard chemotherapy regimens and, instead, possibly more adapted to the non-standard chemotherapy drugs Pictetinib, Taselisib (**Figures 11B, C**).

## Discussion

Programmed cell death is a life-rule for cellular self-repair and regulation, and multiple homeostatic PCD mechanisms ensure the continuity of life activity; however, disordered PCD processes provide necessary assistance for malignant tumor development and metastasis (7). Based on the information we retrieved, the present study was the first to discern novel molecular subtypes in OV and establish prognostic signatures according to 12 PCD signature-related genes.

In this study, we analyzed the influence of PCD on OV progression in the direction of PCD-related genes in OV. 98.2% of OV patients had mutations in PCD-related genes, with TP53 mutations being the most common and prone to co-occurrence between genes. High frequency mutations in TP53 drive high-grade serous ovarian cancers, and furthermore, high frequency TP53 mutations were important molecular phenotypes for the development of some poor prognostic subtypes in cancer (25). The frequency of TP53 mutations in OV patients was 88%.

Tumors distinguished from conventional diseases were characterized by high degree of heterogeneity, and the unique genetic information of individuals was elusive to describe uniformly (26). Traditional pathological staging was based on the histological staging of patients as well as the degree of tissue differentiation. In this study, PCD-related genes based on prognostic traits in OV were subdivided by consistent clustering analysis into four molecular subtypes with different prognosis based on molecular features and exhibiting different immune activities. Functional analysis also explained the different prognosis among molecular subtypes, with the enhanced components of extracellular matrix, collagen-containing extracellular matrix in C1 exacerbating ECM-receptor interaction, PI3K-Akt The increase in the immunoreactive components of MHC protein complex and MHC class II protein complex in C4 enhanced their TME activity. This result was further validated in the TME activity analysis as well.

In this study, we selected DEGs between subtypes for COX analysis and identified 9 genes affecting the prognosis of OV patients. 9-gene composition of Risk Score was significantly effective in assessing the prognosis of OV patients. Patients with low Risk Score had more favorable median survival time and overall survival time and exhibited positive TME environment. The low Risk Score group exhibited high levels of T_cells_CD8, NK_cells_activated, and Macrophages_M1 infiltration, and the high-risk group exhibited high levels of Macrophages_M2 infiltration. CD8+ T cells are the main killer of tumor cells, and factors such as CAF accumulated in TME can lead to CD8+ T cells depletion, which in turn led to immune escape of tumor cells (27). Macrophage M1 was mainly anti-tumor active, and Macrophage M1 could secrete pro-inflammatory cytokines to inhibit tumor growth (28). In contrast, M2 macrophages would stimulate tumor growth by promoting tumor immunosuppression (29). *In vivo* mouse studies revealed that IFN-γ induced the polarization of resting macrophages toward a pro-inflammatory and tumor cytotoxic M1 phenotype to activate anti-tumor immunity and regulate tumor microenvironment with anti-tumor effects (30). Another point to note was that PCD processes were connected with anti-inflammatory responses, while Macrophage M1 and Macrophage M2 were closely associated with inflammatory responses (31). Targeting the direction of Macrophage polarization seems to be a viable direction of research in the early stages of immunotherapy.

Interestingly, Risk Score also had excellent performance in guiding chemotherapy strategies. This study found that patients with high Risk Score were more sensitive to Pictilisib and Taselisib. Pictilisib, also known as GDC-0941, and Taselisib, also known as GDC-0032, both belong to PI3K inhibitors and shown good tumor killing ability in solid tumors (31, 32). Some combinations of Pictilisib and Taselisib were new treatments for some solid tumors. Pictilisib worked by enhancing sensitivity and reducing resistance to temozolomide in GBM cell lines (33). The combination of Pictilisib and Docetaxel enhanced the apoptosis of breast cancer cells and enhanced the anticancer effect of Docetaxel (34). Autophagy signaling was increased in breast cancer cells treated with Taselisib, and chloroquine enhances the antitumor activity of Taselisib in combination with Paclitaxel by inhibiting autophagy signaling (35). The PI3K-Akt signaling pathway was activated in the C1 group in this study, and the proportion of patients with C1 type was also higher in the high Risk Score group. As PI3K inhibitors, Pictilisib and Taselisib may effectively inhibit the activity of PI3K−Akt signaling pathway in patients with high Risk Score and enhanced anti-tumor activity, which might be the reason why the high Risk Score group is sensitive to Pictilisib treatment. In addition, we found that patients in the low-risk Score group exhibited higher TMB. Studies suggested that in lung cancer and bladder cancer, high TMB predicted that patients could benefit from ICB treatment (36). Study also revealed that patients with lung adenocarcinoma with high mutations seemed to be more easily from the PD1/PD-L1 resistant benefit immune therapy (37). In this study, we also found that patients in the low-risk Score group had higher tumor immune cycle activity and immune activation status overall. Immune activation in cancer patients contributed to enhance immune checkpoint gene blockade (38). Our constructed Risk Score appears to be a novel immunotherapy guidance tool.

In conclusion, in this study, we subdivided OV into four subtypes at the molecular level using PCD-related genes as the study target, which can effectively distinguish OV patients with different immune status according to different subtype characteristics. Further, we also established Risk Score, which showed excellent predictive performance in the direction of OV prognosis, TME activity and assessment of immunotherapy and chemotherapy drug selection, providing a research basis for better understanding the impact of PCD on OV progression.

## Data availability statement

The original contributions presented in the study are included in the article/**Supplementary Material**. Further inquiries can be directed to the corresponding author.

## Author contributions

All authors listed have made a substantial, direct, and intellectual contribution to the work and approved it for publication.

## Supplementary material

Supplementary Figure S1 PCD-related genes affecting the prognosis of OV.

Supplementary Figure S2 Venn diagram of DEGs in 4 subtypes.

## References

[1] Stewart C Ralyea C Lockwood S. Ovarian cancer: an integrated review. Semin Oncol Nurs (2019) 35:151–6. doi: 10.1016/j.soncn.2019.02.001 30867104

[2] Kandalaft LE Odunsi K Coukos G. Immunotherapy in ovarian cancer: are we there yet? J Clin Oncol (2019) 37:2460–71. doi: 10.1200/JCO.19.00508 31403857

[3] Sung H Ferlay J Siegel RL Laversanne M Soerjomataram I Jemal A. Global cancer statistics 2020: GLOBOCAN estimates of incidence and mortality worldwide for 36 cancers in 185 countries. CA Cancer J Clin (2021) 71(3):209–49. doi: 10.3322/caac.21660 33538338

[4] Bedoui S Herold MJ Strasser A. Emerging connectivity of programmed cell death pathways and its physiological implications. Nat Rev Mol Cell Biol (2020) 21:678–95. doi: 10.1038/s41580-020-0270-8 32873928

[5] Tang D Kang R Berghe TV Vandenabeele P Kroemer G. The molecular machinery of regulated cell death. Cell Res (2019) 29(5):347–64. doi: 10.1038/s41422-019-0164-5 PMC6796845 30948788

[6] Tang D Chen X Kroemer G. Cuproptosis: a copper-triggered modality of mitochondrial cell death. Cell Res (2022) 32:417–8. doi: 10.1038/s41422-022-00653-7 PMC9061796 35354936

[7] Su Z Yang Z Xu Y Chen Y Yu Q. Apoptosis, autophagy, necroptosis, and cancer metastasis. Mol Cancer (2015) 14:48. doi: 10.1186/s12943-015-0321-5 25743109 PMC4343053

[8] Carneiro BA El-Deiry WS. Targeting apoptosis in cancer therapy. Nat Rev Clin Oncol (2020) 17:395–417. doi: 10.1038/s41571-020-0341-y 32203277 PMC8211386

[9] Zhang Z Zhang Y Xia S Kong Q Li S Liu X. Gasdermin e suppresses tumour growth by activating anti-tumour immunity. Nature (2020) 579(7799):415–20. doi: 10.1038/s41586-020-2071-9 PMC7123794 32188940

[10] Jiang M Qi L Li L Li Y. The caspase-3/GSDME signal pathway as a switch between apoptosis and pyroptosis in cancer. Cell Death Discov (2020) 6:112. doi: 10.1038/s41420-020-00349-0 33133646 PMC7595122

[11] Zou Y Xie J Zheng S Liu W Tang Y Tian W. Leveraging diverse cell-death patterns to predict the prognosis and drug sensitivity of triple-negative breast cancer patients after surgery. Int J Surg (2022) 107:106936. doi: 10.1016/j.ijsu.2022.106936 36341760

[12] Shen W Song Z Zhong X Huang M Shen D Gao P. Sangerbox: a comprehensive, interaction-friendly clinical bioinformatics analysis platform. iMeta (2022) 1(3):e36. doi: 10.1002/imt2.36 PMC10989974 38868713

[13] Wilkerson MD Hayes DN. ConsensusClusterPlus: a class discovery tool with confidence assessments and item tracking. Bioinformatics (2010) 26:1572–3. doi: 10.1093/bioinformatics/btq170 PMC2881355 20427518

[14] Ritchie ME Phipson B Wu D Hu Y Law CW Shi W. Limma powers differential expression analyses for RNA-sequencing and microarray studies. Nucleic Acids Res (2015) 43(7):e47. doi: 10.1093/nar/gkv007 25605792 PMC4402510

[15] Simon N Friedman J Hastie T Tibshirani R. Regularization paths for cox's proportional hazards model *via* coordinate descent. J Stat Softw (2011) 39(5):1–13. doi: 10.18637/jss.v039.i05 PMC4824408 27065756

[16] Kassambara A Kosinski M Biecek P Fabian S. Survminer: drawing survival curves using Ggplot2. R package version 03 (2017) 1.

[17] Liberzon A Birger C Thorvaldsdottir H Ghandi M Mesirov JP Tamayo P. The molecular signatures database (MSigDB) hallmark gene set collection. Cell Syst (2015) 1(6):417–25. doi: 10.1016/j.cels.2015.12.004 PMC4707969 26771021

[18] Hanzelmann S Castelo R Guinney J. GSVA: gene set variation analysis for microarray and RNA-seq data. BMC Bioinf (2013) 14:7. doi: 10.1186/1471-2105-14-7 PMC3618321 23323831

[19] Schubert M Klinger B Klunemann M Sieber A Uhlitz F Sauer S. Perturbation-response genes reveal signaling footprints in cancer gene expression. Nat Commun (2018) 9(1):20. doi: 10.1038/s41467-017-02391-6 29295995 PMC5750219

[20] Yoshihara K Shahmoradgoli M Martinez E Vegesna R Kim H Torres-Garcia W. Inferring tumour purity and stromal and immune cell admixture from expression data. Nat Commun (2013) 4:2612. doi: 10.1038/ncomms3612 24113773 PMC3826632

[21] Chen B Khodadoust MS Liu CL Newman AM Alizadeh AA. Profiling tumor infiltrating immune cells with CIBERSORT. Methods Mol Biol (2018) 1711:243–59. doi: 10.1007/978-1-4939-7493-1_12 PMC5895181 29344893

[22] Hu FF Liu CJ Liu LL Zhang Q Guo AY. Expression profile of immune checkpoint genes and their roles in predicting immunotherapy response. Brief Bioinform (2021) 22(3). doi: 10.1093/bib/bbaa176 32814346

[23] Maeser D Gruener RF Huang RS. oncoPredict: an r package for predicting *in vivo* or cancer patient drug response and biomarkers from cell line screening data. Brief Bioinform (2021) 22. doi: 10.1093/bib/bbab260 PMC8574972 34260682

[24] Gentleman RC Carey VJ Bates DM Bolstad B Dettling M Dudoit S. Bioconductor: open software development for computational biology and bioinformatics. Genome Biol (2004) 5(10):R80. doi: 10.1186/gb-2004-5-10-r80 15461798 PMC545600

[25] Silwal-Pandit L Langerod A Borresen-Dale AL. TP53 mutations in breast and ovarian cancer. Cold Spring Harb Perspect Med (2017) 7. doi: 10.1101/cshperspect.a026252 PMC5204332 27815305

[26] Kroeger PT Jr. Drapkin R. Pathogenesis and heterogeneity of ovarian cancer. Curr Opin Obstet Gynecol (2017) 29:26–34. doi: 10.1097/GCO.0000000000000340 27898521 PMC5201412

[27] Raskov H Orhan A Christensen JP Gogenur I. Cytotoxic CD8(+) T cells in cancer and cancer immunotherapy. Br J Cancer (2021) 124(2):359–67. doi: 10.1038/s41416-020-01048-4 PMC7853123 32929195

[28] Daldrup-Link HE Golovko D Ruffell B Denardo DG Castaneda R Ansari C. MRI Of tumor-associated macrophages with clinically applicable iron oxide nanoparticles. Clin Cancer Res (2011) 17(17):5695–704. doi: 10.1158/1078-0432.CCR-10-3420 PMC3166957 21791632

[29] Pan Y Yu Y Wang X Zhang T. Tumor-associated macrophages in tumor immunity. Front Immunol (2020) 11:583084. doi: 10.3389/fimmu.2020.583084 33365025 PMC7751482

[30] Cao Q Yan X Chen K Huang Q Melancon MP Lopez G. Macrophages as a potential tumor-microenvironment target for noninvasive imaging of early response to anticancer therapy. Biomaterials (2018) 152:63–76. doi: 10.1016/j.biomaterials.2017.10.036 29111494 PMC5693615

[31] Mehla K Singh PK. Metabolic regulation of macrophage polarization in cancer. Trends Cancer (2019) 5:822–34. doi: 10.1016/j.trecan.2019.10.007 PMC7187927 31813459

[32] Olivero AG Heffron TP Baumgardner M Belvin M Ross LB Blaquiere N. Abstract DDT02-01: discovery of GDC-0032: a beta-sparing PI3K inhibito r active against PIK3CA mutant tumors. Cancer Res (2013) 73(8_Supplement):DDT02–1-DDT-1. doi: 10.1158/1538-7445.AM2013-DDT02-01

[33] Shi F Guo H Zhang R Liu H Wu L Wu Q. The PI3K inhibitor GDC-0941 enhances radiosensitization and reduces chemoresistance to temozolomide in GBM cell lines. Neuroscience (2017) 346:298–308. doi: 10.1016/j.neuroscience.2017.01.032 28147244

[34] Wallin JJ Guan J Prior WW Lee LB Berry L Belmont LD. GDC-0941, a novel class I selective PI3K inhibitor, enhances the efficacy of docetaxel in human breast cancer models by increasing cell death *in vitro* and in vivo. Clin Cancer Res (2012) 18(14):3901–11. doi: 10.1158/1078-0432.CCR-11-2088 22586300

[35] Cocco S Leone A Roca MS Lombardi R Piezzo M Caputo R. Inhibition of autophagy by chloroquine prevents resistance to PI3K/AKT inhibitors and potentiates their antitumor effect in combination with paclitaxel in triple negative breast cancer models. J Transl Med (2022) 20(1):290. doi: 10.1186/s12967-022-03462-z 35761360 PMC9235112

[36] Chan TA Yarchoan M Jaffee E Swanton C Quezada SA Stenzinger A. Development of tumor mutation burden as an immunotherapy biomarker: utility for the oncology clinic. Ann Oncol (2019) 30(1):44–56. doi: 10.1093/annonc/mdy495 30395155 PMC6336005

[37] Dantoing E Piton N Salaun M Thiberville L Guisier F. Anti-PD1/PD-L1 immunotherapy for non-small cell lung cancer with actionable oncogenic driver mutations. Int J Mol Sci (2021) 22(12):6288. doi: 10.3390/ijms22126288 34208111 PMC8230861

[38] Tang B Wang Y Xu W Zhu J Weng Q Chen W. Macrophage xCT deficiency drives immune activation and boosts responses to immune checkpoint blockade in lung cancer. Cancer Lett (2023) 554:216021. doi: 10.1016/j.canlet.2022.216021 36455758

