# Supplementary figures and images for: Assessing of programmed cell death gene signature for predicting ovarian cancer prognosis and treatment response
Source: Front Endocrinol (Lausanne). 2023 Jun 5;14:1182776. doi: 10.3389/fendo.2023.1182776 (PMC10277615; doi:10.3389/fendo.2023.1182776)

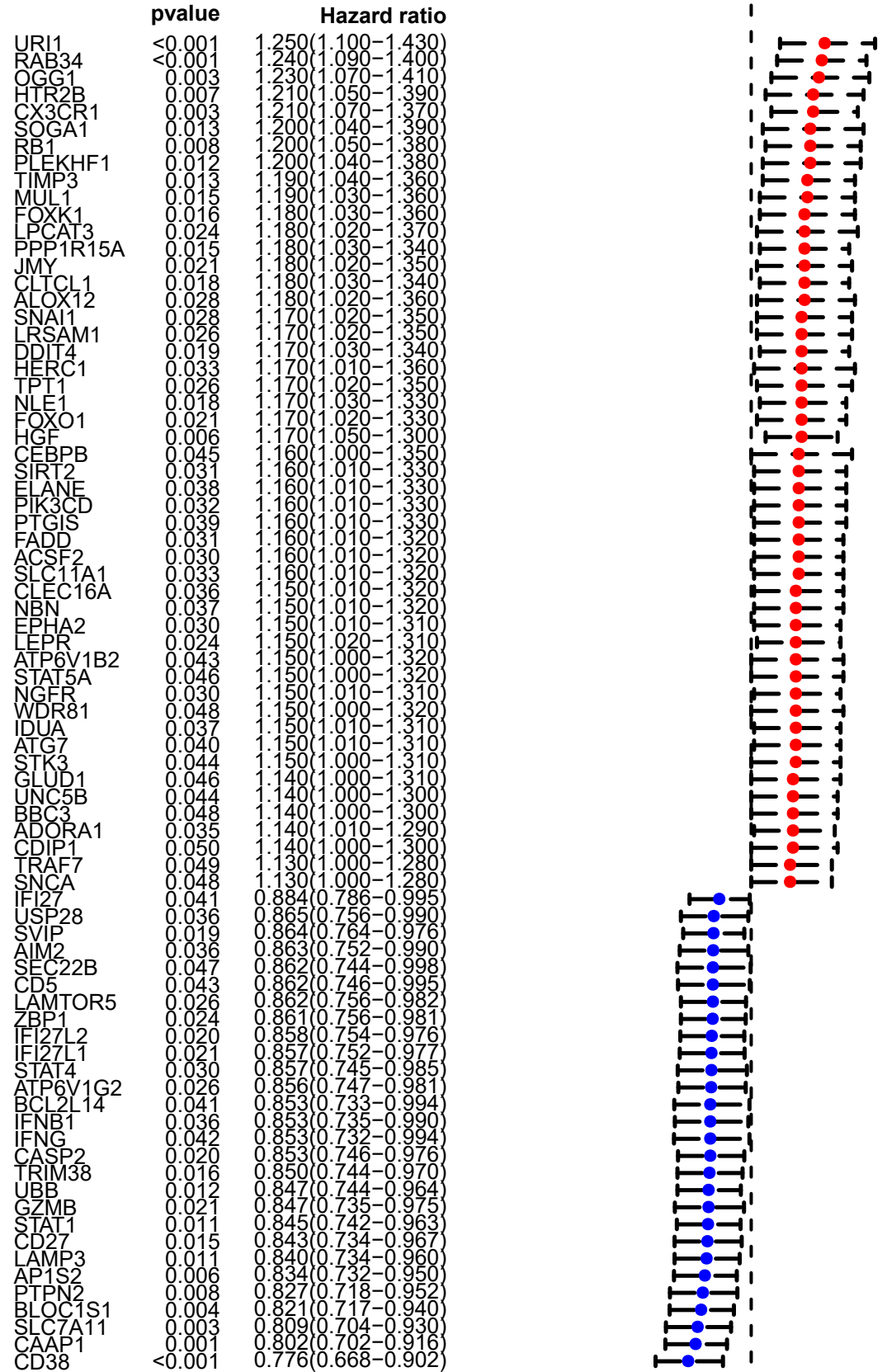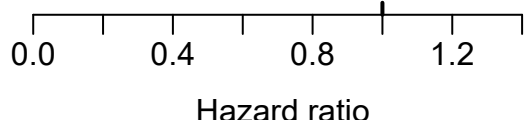

Supplement: Supplementary Figure S1 — PCD-related genes affecting the prognosis of OV. [file Image_1.pdf]

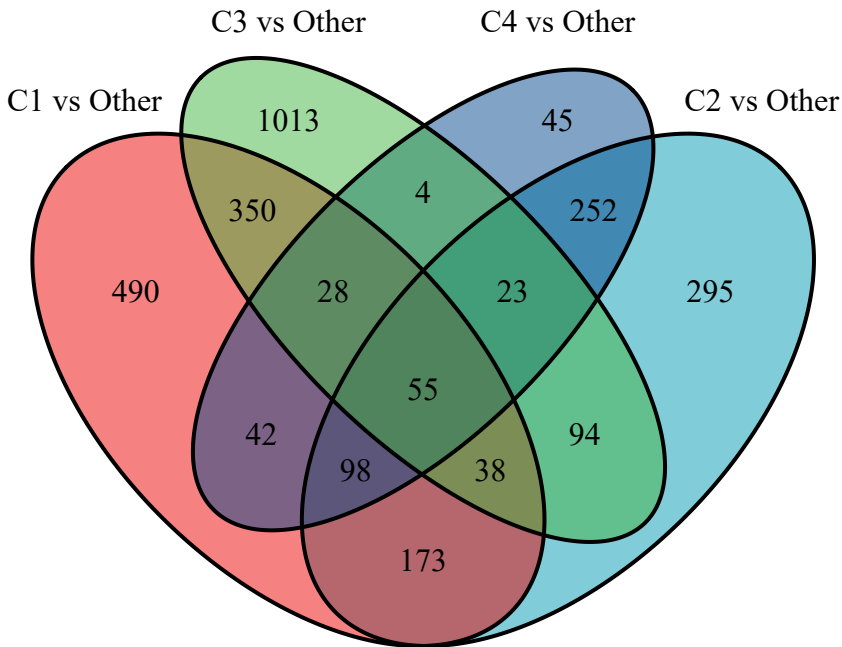

Supplement: Supplementary Figure S2 — Venn diagram of DEGs in 4 subtypes. [file Image_2.pdf]
